# Polymorphism and plasma levels of apolipoprotein E and the risk of aneurysmal subarachnoid hemorrhage in a Chinese population: a case-control study

**DOI:** 10.1186/s12944-018-0755-z

**Published:** 2018-05-16

**Authors:** Xiaofeng Liu, Weiwu Zhan, Qiumei Wu, Fengqing Wang, Bin Yang, Qishui Ou

**Affiliations:** 1 Department of Laboratory Medicine, the First Affiliated Hospital of Fujian Medical University, No.20 Chazhong Road, Fuzhou, 350005 China; 2 Gene Diagnostic Laboratory, the First Affiliated Hospital of Fujian Medical University, No.20 Chazhong Road, Fuzhou, 350005 China

**Keywords:** Subarachnoid hemorrhage, Aneurysms, Apolipoprotein E, Gene polymorphism, Blood lipids, Chinese Han population

## Abstract

**Background:**

Aneurysmal subarachnoid hemorrhage (aSAH) is the most common types of subarachnoid hemorrhage, which is a critical clinical problem with high morbidity, mortality, and economic impact. Recent studies have shown that APOE was a genetic risk factor of aSAH, however, the studies lack consistent conclusions and the evidence from Chinese Han population is rare.

**Objective:**

To determine the relationship between APOE polymorphism and the incidence of aSAH in Chinese Fujian Han population and explore the possible mechanism of ApoE in the pathogenesis of aSAH.

**Methods:**

A total of 131 patients newly diagnosed with aSAH were selected as aSAH group and 137 healthy subjects were selected as the control group. All the samples were analyzed for blood lipids and serum ApoE levels, and ApoE genotype was determined by a commercial chip and further confirmed with Sanger sequencing. An adjusted multivariate logistic regression analysis was carried out to estimate the effects of APOE polymorphism on the risk of aSAH.

**Results:**

Compared with the controls, the serum TC, HDL-C and ApoA1 levels in aSAH were significantly lower: TC (4.52 ± 1.38 vs. 5.11 ± 0.86 mmol/L, *P* < 0.001), HDL-C (1.23 ± 0.46 vs. 1.44 ± 0.32 mmol/L, *P* < 0.001) and ApoA1 (1.20 ± 0.32 vs. 1.38 ± 0.25 g/L, *P* < 0.001). The distribution of ε2/ε3 genotype (19.08% vs. 9.49%, *P* = 0.038) and ε2 allele frequency (11.07% vs. 5.84%, *P* = 0.039) was significantly higher in aSAH than the healthy controls. The multivariate logistic regression identified that ApoE ε2 allele was independently associated with aSAH (OR = 2.083; and 95% CI = 1.045-4.153, *P* = 0.037). The serum ApoE in aSAH were significantly higher than controls (53.03 ± 24.64 vs. 45.06 ± 12.84 mg/L, *P* = 0.010).

**Conclusion:**

APOE polymorphism might be associated with the incidence of aSAH in Chinese Fujian Han population. ApoE ε2 may be a risk factor for the incidence of aSAH, which may be related with the impacts of ApoE genotypes for the serum lipids, especially for the plasma levels of ApoE.

## Background

Subarachnoid hemorrhage (SAH) is a neurological emergency and a severe subtype of stroke with high morbidity and mortality, which occurs at younger ages than ischemia stroke or intracerebral hemorrhage. It is reported that about 85% of SAH is due to the rupture of an intracranial aneurysm, leading to aneurysmal subarachnoid hemorrhage (aSAH) [1]. As the incidence of aSAH is urgent and dangerous, the epidemiological data have shown an estimated 10% of aSAH patients die before getting medical treatment [2]. Meanwhile, it is reported that with the 3-month mortality rate reaching as high as 47% to 49%, and half of all survivors are left severely disabled [3–5]. Therefore, until now the treatment of aSAH in clinical remains unpredictable and the rate of mortality and disability is still high.

Accumulating evidence have shown that aSAH was a multifactorial disorder associated with genetic and environmental factors [6]. Many studies have evaluated modifiable risk factors for aSAH, including age, female sex, hypertension, and tobacco use [7, 8]. Meanwhile, a lot of evidence have suggested that genetic predisposition is also involved in aSAH [9]. So, the discovery of the effects of gene polymorphisms in the course of aSAH, and development of personalized diagnosis and therapy might show attractive prospect in the management of aSAH.

Recently, a number of evidences have suggested that apolipoprotein E (APOE for gene, ApoE for protein) is a candidate gene that is associated with aSAH [10–13]. In humans, APOE is located on 19q13 and has three common alleles: *ε*2, *ε*3, and *ε*4, which form six ApoE genotypes: *ε*2/*ε*2, *ε*2/*ε*3, *ε*2/*ε*4, *ε*3/*ε*3, *ε*3/*ε*4, and *ε*4/*ε*4. The *ε*3/*ε*3 phenotype has the widest distribution in population, refer as “wild type” [14]. Human APOE codes a 34-kDa protein with 299 amino acids (ApoE), composed with three major isoforms (ApoE2, ApoE3 and ApoE4), and participated in cholesterol and lipid transport [15–17]. Especially, ApoE plays a critical role in redistributing lipids among central nervous system, repairing injured neurons, neurite outgrowth, and glucose use by neurons and glial cells [18, 19].

It has been identified that the ApoE ε4 allele is a major risk factor for Alzheimer’s disease [20–22]. Alternatively, presence of the ε4 allele has been associated with poor outcomes after traumatic brain injury [23]. Recently, the association between the APOE polymorphism and risk of aSAH has been investigated in several studies among different populations, however, the conclusion from these studies were conflicting [13, 24, 25]. Furthermore, most of evidences were from Caucasian populations in Europe and USA, the direct evidence from Chinese Han population is relatively poor [26].

The primary aim of this study was to demonstrate whether APOE polymorphism is an important determinant of aSAH in Chinese Han population in Fujian province, which is located in the south-east of China. Secondarily, we further explore the possible mechanism whether the ApoE genotypes influence the incidence of aSAH is associated with the serum lipids, especially the concentration of plasma ApoE.

## Methods

### Study subjects and design

The present study consisted of 131 aSAH patients who were newly diagnosed in the First Affiliated Hospital of Fujian Medical University (Fuzhou, China) from June 2016 to August 2017. All patients with aSAH were eligible for the following criteria: 48 h of onset, with headache, nausea, vomiting, meningeal irritation and other typical clinical manifestations; and the aneurysm were confirmed by digital subtraction angiography (DSA), computed tomography angiography (CTA) or surgery. Meanwhile, 137 age- and gender-matched healthy adults, who were seeking routine health check-up at the First Affiliated Hospital of Fujian Medical University, were used as control subjects. The others who were suffering from chronic liver disease, kidney disease, other tumors and shorter survival time were excluded from this study.

The study protocol was approved by the ethics committees of the First Affiliated Hospital of Fujian Medical University and was performed in accordance with the Declaration of Helsinki. Written informed consent was obtained from all of the participants prior to the study.

### Blood lipid assay

Peripheral venous blood (3~ 5 mL) was collected in serum from participants after at least 12 h of fasting, and serum was immediately separated by a 10 min centrifugation at 1500 g. The serum samples were stored at − 80 °C until the analysis. Serum lipid levels including serum total cholesterol (TC), triglyceride (TG), low density lipoprotein cholesterol (LDL-C), high density lipoprotein cholesterol (HDL-C), apolipoproteinA1 (ApoA1), apolipoprotein B (ApoB), and apolipoprotein E (ApoE) were examined by automatic biochemistry analyzer (Siemens, ADVIA2400).

### Apolipoprotein E genotype analysis

Genomic DNA was extracted from venous blood according to the manufacturer’s recommendations (Qiagen). The concentration of DNA was determined using a NanoDrop 1000 spectrophotometer (Thermo Fisher). DNA samples were frozen at − 20 °C until processed. For the determination the ApoE genotype, a commercial ApoE genotyping chip (Sinochips, China) was performed according to the protocols provided by the manufacture.

Meanwhile, we further confirmed the ApoE genotype by Sanger sequencing, and the PCR primers were designed based on the GenBank reference as follow: sense, GACCATGAAGGAGTTGAAGGCCTAC; antisense, CTCGCGGGCCCCGGCCTGGTA.

### Statistical analysis

Statistical analysis was performed with SPSS 18.0 software (Chicago, USA). The data were expressed as the mean ± standard deviation (SD) for numerical variables, or as the number (%) for categorical variables. The Hardy-Weinberg equilibrium was estimated in healthy controls. The independent t-test or the Chi-square test was used to compare outcomes. A multivariate logistic regression analysis adjusted for age and gender was carried out using aSAH as dependent variable, the data was showed as the odds ratios (ORs) and 95% confidence intervals (CIs). All tests of significance were two-tailed, and *P* < 0.05 was considered statistically significant.

## Results

### Clinical and laboratory characteristics of studying subjects

In our present study, 159 patients with aSAH within 48 h of onset were enrolled, 19 patients were excluded because their clinical data were not gotten completely. Nine patients who did not verified the responsible vascular lesion were also not included. The remaining 131 patients were recruited as aSAH group finally.

The general characteristics and serum lipids in this study are shown in Table 1. As to general characteristics, there were 268 subjects which included 131 patients with aSAH (55 males and 76 females) as aSAH group and 137 age- and gender-matched healthy adults (57 males and 80 females) as control group. The average age of the aSAH group was 54.65 years, with a standard deviation of 13.53, and those of healthy controls was 55.23 years, with a standard deviation of 10.44. There were no significant difference of age, sex, smoking, drinking, hypertension and BMI between the two groups (All *P*>0.05).

**Table 1:** Clinical and laboratory characteristics of patients with aSAH and healthy controls

	aSAH(*n* = 131)	Controls(*n* = 137)	*P* value
Age (years)	54.65 ± 13.53	55.23 ± 10.44	0.688
Sex-No (male %)	41.98(55/131)	41.61(57/137)	0.928
BMI (kg/m2)	24.84 ± 3.79	24.16 ± 3.39	0.744
Smoking (%)	26.43(37/131)	23.36(32/137)	0.581
Drinking (%)	15.27(20/131)	18.98(26/137)	0.421
Hypertension (%)	43.51(57/131)	38.69(53/137)	0.422
TC (m mol/L)	4.52 ± 1.38	5.11 ± 0.86	< 0.001^*^
TG (m mol/L)	1.23 ± 0.77	1.29 ± 0.75	0.566
HDL-C (m mol/L)	1.23 ± 0.46	1.44 ± 0.32	< 0.001^*^
LDL-C (m mol/L)	3.19 ± 1.47	3.27 ± 0.82	0.625
ApoA1 (g/L)	1.20 ± 0.32	1.38 ± 0.25	< 0.001^*^
ApoB (g/L)	0.93 ± 0.31	0.99 ± 0.21	0.075

Data are expressed as the means ± standard deviation, except for sex, smoking, drinking and hypertension which is expressed as the number (%). *TC* Total cholesterol, *TG* Triglyceride, *HDL-C* High density lipoprotein cholesterol, *LDL-C* Low density lipoprotein cholesterol, *ApoA1* Apolipoproteina1, *ApoB* Apolipoprotein B

*P* value, 2-sample independent t-test, or chi-squared test as appropriate. ^*^P < 0.001, compared with controls

As to the levels of serum lipids (including TC, TG, HDL-C, LDL-C, ApoA1, and ApoB), there were no significant differences in the levels of TG, LDL-C and ApoB between the aSAH groups and control groups (all *P* > 0.05). However, the concentration of TC (aSAH groups: 4.52 ± 1.38 mmol/L; control groups: 5.11 ± 0.86 mmol/L), HDL-C (aSAH groups: 1.23 ± 0.46 mmol/L; control groups: 1.44 ± 0.32 mmol/L), and ApoA1 (aSAH group: 1.20 ± 0.32 g/L; control groups: 1.38 ± 0.25 g/L) were significant differences between two groups (all *P* < 0.001). As shown in Table 1, the serum TC,HDL-C and ApoA1 levels in aSAH were significantly lower than those in the controls.

### APOE polymorphism distribution in aSAH and controls

The genotype and allele frequencies between patients with aSAH and healthy controls are shown in Table 2. The ApoE allelic frequency in controls was consistent with Hardy-Weinberg equilibrium (*P* > 0.05). Six common genotypes of human APOE were identified in our study. Among them, the genotype ε3/ε3 had the largest proportion both in aSAH groups and control groups, and then ε2/ε3 and ε3/ε4, while ε2/ε2, ε4/ε4 were the least, which is comparable to the values found in other studies performed in Asian populations [27, 28]. We further observed the differences in the distribution of the 6 common genotypes in the two groups, we found that the distribution of ε2/ε3 genotype was higher in the aSAH group than the healthy control group (aSAH group: 19.08%; control groups: 9.49%, *P* < 0.05). Although the ratio of ε3/ε4 in aSAH was lower, there was no statistically significant difference compared with controls (*P* > 0.05).

**Table 2:** Genotypes and allelic frequency of APOE in aSAH group and control group

		aSAH(*n* = 131)		Controls(*n* = 137)		*P* value
		No	%	No	%	
Genotype	ε2/ε2	1	0.76	1	0.73	0.941
	ε2/ε3	25	19.08	13	9.49	0.038*
	ε2/ε4	2	1.53	1	0.73	0.606
	ε3/ε3	90	68.71	100	72.99	0.440
	ε3/ε4	12	9.16	20	14.60	0.300
	ε4/ε4	1	0.76	2	1.46	0.999
Allele	ε2	29	11.07	16	5.84	0.039^#^
	ε3	217	82.82	232	84.67	0.562
	ε4	16	6.11	26	9.49	0.204

^*^Genotypes frequency, Chi^2^, *P* < 0.05

^#^Allelic frequency: ε2 (Chi^2^, *P* < 0.05)

Then, we also observed the allele frequencies between the two groups, the ε3 allele was greatest (82.82%), while those of ε2 and ε4 were 11.07 and 6.11%, respectively in aSAH patients. Meanwhile, ε3 allele was 84.67%, and ε2 and ε4 were 5.84 and 9.49% in controls. We found that the frequency of ε2 in aSAH group was higher than that in control group (aSAH group: 11.07%; control groups: 5.84%, *P* < 0.05), indicated that ApoE ε2 may be play some roles in aSAH.

### The association of APOE polymorphism with the incidence of aSAH

Using the multivariate logistic regression analysis adjusted for age and gender, we further explore the correlation between APOE Polymorphism and the incidence of aSAH.

As shown in Table 3, after adjusted for the age and gender, there was no significant difference in the incidence of aSAH between the allele ε4 and ε3 (OR = 0.650, 95% CI = 0.308-1.371, and *P* = 0.258), while, allele ε2 was significantly increased compared to allele ε3 in aSAH (OR = 2.083, 95% CI = 1.045-4.153, and *P* = 0.037), suggesting that ε2 is a risk factor for aSAH compared with ε3.

**Table 3:** Odds ratios and 95% confidence interval for aSAH under three major genetic models

	*P* value	OR(95%CI)
ε2 versus ε3	0.037^*^	2.083(1.045, 4.153)
ε4 versus ε3	0.258	0.650(0.308, 1.371)

*P* value was obtained from the logistic regression after adjustment for age and gender. *OR* Odds ratio, *CI* Confidence Interval. ^*^P < 0.05

### The association of plasma levels of ApoE with aSAH

As shown in Table 4, the differences of serum ApoE between the two groups were analyzed by 2-sample independent t-test. There was a statistically significant difference between the two groups (*P* < 0.05), the levels of ApoE in aSAH (53.03 ± 24.64 mg/L) was higher than those in controls (45.06 ± 12.84 mg/L).

**Table 4:** the plasma levels of ApoE in aSAH group and control group

	aSAH(*n* = 131)	Controls(*n* = 137)	*P* value
ApoE(mg/L)	53.03 ± 24.64	45.06 ± 12.84	0.010^*^

Data are expressed as the means ± standard deviation, *P* value for independent t-test, 2-tailed. ^*^P < 0.05, compared with controls

Meanwhile, as shown in Table 5, in order to explore the effects of genotype on serum ApoE, we further analyzed the plasma levels of ApoE in the three common genotypes (ε2/ε3, ε3/ε3 and ε3/ε4) in aSAH patients (The proportion of other three genotypes was too low, not yet compared). The data showed the plasma levels of ApoE in the three common genotypes were as follow: ε2/ε3 > ε3/ε3 > ε3/ε4, but there was not yet significant difference (all *P* > 0.05).

**Table 5:** the plasma levels of ApoE in aSAH group under three genetic models

	ε2/ε3	ε3/ε3	ε3/ε4
ApoE(mg/L)	56.81 ± 17.09	54.17 ± 26.32	37.47 ± 22.63
*P* value	0.742	–	0.141

Data are expressed as the means ± standard deviation, *P* value for independent t-test, 2-tailed

## Discussion

As we known, aSAH is a critical clinical problem with less chance for patient recovery and survival even after surgical management and medication. So, the discovery of genetic predisposition of aSAH show attractive prospects for improve its diagnosis and treatment, and making medical decisions in clinical practice by personalized medicine.

It has been suggested that APOE polymorphism play a critical role in the incidence and prognosis of aSAH, which has been shown in various studies among different population [10–12]. However, these studies have failed to reach a definitive conclusion. Furthermore, most of evidences were from Caucasian populations, the direct evidence in Chinese Han population is relatively poor.

A recently meta-analysis of 9 studies, which was mainly including Caucasian and Asian population, have showed that there was a significant association between APOE genotype and aSAH, and ε2 allele was a risk factor for aSAH [24]. In our present study, we found the distribution of ε2/ε3 genotype in aSAH was higher than the healthy controls, and allele frequencies of ApoE ε2 in aSAH was also higher than controls. Latter, using the multivariate logistic regression analysis, we found that ApoE ε2 allele was significantly increased compared to ε3 in aSAH (OR = 2.083, 95% CI = 1.045-4.153, *P* = 0.037), suggesting that ε2 allele is a risk factor for aSAH, which was consistent with the result of the previous meta-analysis [24] and the study of Liu et al. [12].

As we known, ApoE is a plasma glycoprotein and play an important role in the transport of cholesterol and other lipids. A number of studies have shown that ApoE genotypes could influence the levels of serum lipids [29–31]. Meanwhile, recent studies have showed that the serum lipids participated in the pathogenesis of aSAH [32, 33]. In this study, we also determined the serum lipids in aSAH and healthy controls, the data showed that the levels of TC, HDL-C and ApoA1 in aSAH were significantly lower than those in healthy controls (all *P* < 0.05). It is indicated that a decrease in plasma TC, HDL-C and ApoA1 levels is associated with the occurrence of aSAH. Our results was consistent with the other studies that the carrier of ε2 allele, is associated with the lower total cholesterol compared with ε3 and ε4 [34].

ApoE is not only present in plasma, but also in the central nervous systems. It has reported that ApoE in the central nervous systems plays a critical role in redistributing lipids for normal lipid homeostasis, repairing injured neurons, neurite outgrowth, synaptic plasticity, which acts as a neurotrophic, protective and repair functions [18]. Meanwhile, recent studies have reported that plasma ApoE is an important regulator of TC, LDL and other lipid metabolic processes [35, 36]. In current study, we further detected the serum ApoE concentration, our data showed that the plasma ApoE in the aSAH was significantly higher than that in the healthy controls (*P* < 0.01). Although no statistically differences in serum ApoE concentrations were observed for the various ApoE genotypes, the trends were observed that the serum ApoE of the ε2/ε3 genotype was higher than that of the ε3/ε3 genotype in aSAH group. It was indicated that the presence of allele ε2 may increase the plasma ApoE content, which may be related with the less effective of ApoE ε2 on neuroprotective function during stress or injury [37].

Although the molecular mechanisms of the association between ApoE and aneurysms are not fully understood, they are becoming increasingly clear. It has been reported that low serum HDL-C is strongly correlated with enhanced status of inflammation, endothelial activation and oxidative stress, which have a greater chance of endothelial weakening in intracerebral arteries [38, 39]. Meanwhile, low serum cholesterol levels were at an increased risk of intracerebral hemorrhage [40]. Furthermore, as we mentioned above, ApoE is a key regulator of the lipid metabolic processes, participated in the dyslipidemia in aSAH. These direct evidence suggested that the serum low HDL-C and TC, which had been observed in our present study, could be increase the endothelial weakening in intracerebral arteries, and in turn enhance the risk of aneurysms rupture and intracerebral hemorrhage. This may be a possible mechanism of ApoE involved in the aneurysms and aSAH.

Furthermore, it should be mentioned that there were some controversial results with our present study on the correlation of APOE polymorphism and aSAH. First of all, a case-control study of Tang et al. from a Beijing Chinese Han population of aSAH suggested that there was no association among APOE polymorphism with the incidence of aSAH [26]. Secondly, a meta-analysis of Zhao et al. suggested that no association of APOE gene polymorphism and SAH susceptibility [41]. In addition, a meta-analysis of Chen et al. found that the ApoE ε4 carriers showed a significantly elevated risk of developing SAH in the Chinese population, which may be a risk factor for SAH [25].

As to the different results, there are some key factors should be mentioned, such as: (1) geographical area difference, (2) the bias in patients selection, (3) the population size and statistical processing, (4) the differences of objective case: some studies used the overall SAH as the objective case, including the traumatic or spontaneous SAH and so on.

For our current study, we focused on the Chinese Fujian Han population, which was located on the southeast of China. Moreover, every patient of aSAH was confirmed by DSA, CTA or surgery. Meanwhile, the ApoE genotype was determined by commercial ApoE genotyping chip (Sinochips, China), which had get the Certification of CFDA (China Food and Drug Administration) and widely used in many hospital in China, and further confirmed by Sanger sequencing.

### Limitations

However, there were also some limitations in our current study. Firstly, the effects of diet on lipid profile in this study should be considered. A lot of studies have reported that the nutraceuticals and functional substances contained in food, such as proanthocyanidins, resveratrol, red wine, and fish oil, maybe have protective effect on vascular system and reduced the overall cardiovascular risk induced by dyslipidemia [42]. Secondly, the patients of aSAH in our study were all come from one hospital which may be a selection bias. Thirdly, those who died or give up during emergency treatment were not included, which could cause Neyman bias. Furthermore, the size of the cases admitted in this study was relatively small. These limitations make us be careful on drawing the conclusions.

## Conclusions

In conclusion, our study have suggested that there was a significant correlation between serum lipids and aSAH, and APOE polymorphism might be associated with the incidence of aSAH in Chinese Fujian Han population and the ApoE ε2 allele was a risk factor for the incidence of aSAH. The serum ApoE was significant higher in aSAH compared with the healthy controls. The mechanisms of ApoE ε2 in the incidence of aSAH may be related with the impacts of ApoE genotypes for the serum lipids. Additional studies of larger population size are needed to confirm this finding.

## References

[1] Macdonald RL, Schweizer TA (2017). Spontaneous subarachnoid haemorrhage. Lancet.

[2] Wijdicks EF, Kallmes DF, Manno EM, Fulgham JR, Piepgras DG (2005). Subarachnoid hemorrhage: neurointensive care and aneurysm repair. Mayo Clin Proc.

[3] de Rooij NK, Linn FH, van der Plas JA, Algra A, Rinkel GJ (2007). Incidence of subarachnoid haemorrhage: a systematic review with emphasis on region, age, gender and time trends. J Neurol Neurosurg Psychiatry.

[4] Broderick JP, Brott TG, Duldner JE, Tomsick T, Leach A (1994). Initial and recurrent bleeding are the major causes of death following subarachnoid hemorrhage. Stroke.

[5] Katati MJ, Santiago-Ramajo S, Perez-Garcia M, Meersmans-Sanchez Jofre M, Vilar-Lopez R, Coin-Mejias MA, Caracuel-Romero A, Arjona-Moron V (2007). Description of quality of life and its predictors in patients with aneurysmal subarachnoid hemorrhage. Cerebrovasc Dis.

[6] Peck G, Smeeth L, Whittaker J, Casas JP, Hingorani A, Sharma P (2008). The genetics of primary haemorrhagic stroke, subarachnoid haemorrhage and ruptured intracranial aneurysms in adults. PLoS One.

[7] Andreasen TH, Bartek J, Andresen M, Springborg JB, Romner B (2013). Modifiable risk factors for aneurysmal subarachnoid hemorrhage. Stroke.

[8] Feigin VL, Rinkel GJ, Lawes CM, Algra A, Bennett DA, van Gijn J, Anderson CS (2005). Risk factors for subarachnoid hemorrhage: an updated systematic review of epidemiological studies. Stroke.

[9] Rosalind Lai PM, Du R (2015). Role of genetic polymorphisms in predicting delayed cerebral ischemia and radiographic vasospasm after aneurysmal subarachnoid hemorrhage: a Meta-analysis. World Neurosurg.

[10] Csajbok LZ, Nylen K, Ost M, Blennow K, Zetterberg H, Nellgard P, Nellgard B (2016). Apolipoprotein E polymorphism in aneurysmal subarachnoid haemorrhage in West Sweden. Acta Neurol Scand.

[11] Kaushal R, Woo D, Pal P, Haverbusch M, Xi H, Moomaw C, Sekar P, Kissela B, Kleindorfer D, Flaherty M (2007). Subarachnoid hemorrhage: tests of association with apolipoprotein E and elastin genes. BMC Med Genet.

[12] Liu H, Mao P, Xie C, Xie W, Wang M, Jiang H (2016). Apolipoprotein E polymorphism and the risk of intracranial aneurysms in a Chinese population. BMC Neurol.

[13] Mineharu Y, Inoue K, Inoue S, Yamada S, Nozaki K, Takenaka K, Hashimoto N, Koizumi A (2006). Association analysis of common variants of ELN, NOS2A, APOE and ACE2 to intracranial aneurysm. Stroke.

[14] Mahley RW, Rall SC (2000). Apolipoprotein E: far more than a lipid transport protein. Annu Rev Genomics Hum Genet.

[15] Mahley RW (1988). Apolipoprotein E: cholesterol transport protein with expanding role in cell biology. Science.

[16] Mahley RW, Huang Y (2012). Apolipoprotein e sets the stage: response to injury triggers neuropathology. Neuron.

[17] Bu G (2009). Apolipoprotein E and its receptors in Alzheimer's disease: pathways, pathogenesis and therapy. Nat Rev Neurosci.

[18] Mahley RW (2017). Apolipoprotein E: remarkable protein sheds light on cardiovascular and neurological diseases. Clin Chem.

[19] Mahley RW (2016). Apolipoprotein E. from cardiovascular disease to neurodegenerative disorders. J Mol Med (Berl).

[20] Corder EH, Saunders AM, Strittmatter WJ, Schmechel DE, Gaskell PC, Small GW, Roses AD, Haines JL, Pericak-Vance MA (1993). Gene dose of apolipoprotein E type 4 allele and the risk of Alzheimer's disease in late onset families. Science.

[21] Strittmatter WJ, Saunders AM, Schmechel D, Pericak-Vance M, Enghild J, Salvesen GS, Roses AD (1993). Apolipoprotein E: high-avidity binding to beta-amyloid and increased frequency of type 4 allele in late-onset familial Alzheimer disease. Proc Natl Acad Sci U S A.

[22] Liu CC, Liu CC, Kanekiyo T, Xu H, Bu G (2013). Apolipoprotein E and Alzheimer disease: risk, mechanisms and therapy. Nat Rev Neurol.

[23] Teasdale GM, Murray GD, Nicoll JA (2005). The association between APOE epsilon4, age and outcome after head injury: a prospective cohort study. Brain.

[24] Arati S, Sibin MK, Bhat DI, Narasingarao KV, Chetan GK (2016). Polymorphisms of apolipoprotein E and aneurysmal subarachnoid haemorrhage: a meta-analysis. Meta Gene.

[25] Chen C, Hu Z (2016). ApoE polymorphisms and the risk of different subtypes of stroke in the Chinese population: a comprehensive Meta-analysis. Cerebrovasc Dis.

[26] Tang J, Zhao J, Zhao Y, Wang S, Chen B, Zeng W (2003). Apolipoprotein E epsilon4 and the risk of unfavorable outcome after aneurysmal subarachnoid hemorrhage. Surg Neurol.

[27] Liu HC, Hong CJ, Wang SJ, Fuh JL, Wang PN, Shyu HY, Teng EL (1999). ApoE genotype in relation to AD and cholesterol: a study of 2,326 Chinese adults. Neurology.

[28] Hallman DM, Boerwinkle E, Saha N, Sandholzer C, Menzel HJ, Csazar A, Utermann G (1991). The apolipoprotein E polymorphism: a comparison of allele frequencies and effects in nine populations. Am J Hum Genet.

[29] Xia Y, Sass C, Shen X, Siest G, Visvikis S (2000). Associations of apolipoprotein E concentration and polymorphism with lipids and apolipoprotein levels in Chinese from Beijing and shanghai. Clin Chem Lab Med.

[30] Han S, Xu Y, Gao M, Wang Y, Wang J, Liu Y, Wang M, Zhang X (2016). Serum apolipoprotein E concentration and polymorphism influence serum lipid levels in Chinese Shandong Han population. Medicine (Baltimore).

[31] Shin MH, Kim HN, Cui LH, Kweon SS, Park KS, Heo H, Nam HS, Jeong SK, Chung EK, Choi JS (2005). The effect of apolipoprotein E polymorphism on lipid levels in Korean adults. J Korean Med Sci.

[32] Tokuda Y, Stein GH (2005). Serum lipids as protective factors for subarachnoid hemorrhage. J Clin Neurosci.

[33] Lindbohm JV, Kaprio J, Korja M (2016). Cholesterol as a risk factor for subarachnoid hemorrhage: a systematic review. PLoS One.

[34] Liang S, Pan M, Geng H, Chen H, Gu L, Qin X, Qian J, Zhu J, Liu C (2009). Apolipoprotein E polymorphism in normal Han Chinese population: frequency and effect on lipid parameters. Mol Biol Rep.

[35] Huang Y, Mahley RW (2014). Apolipoprotein E: structure and function in lipid metabolism, neurobiology, and Alzheimer's diseases. Neurobiol Dis.

[36] Schiele F, De Bacquer D, Vincent-Viry M, Beisiegel U, Ehnholm C, Evans A, Kafatos A, Martins MC, Sans S, Sass C (2000). Apolipoprotein E serum concentration and polymorphism in six European countries: the ApoEurope project. Atherosclerosis.

[37] Dong LM, Parkin S, Trakhanov SD, Rupp B, Simmons T, Arnold KS, Newhouse YM, Innerarity TL, Weisgraber KH (1996). Novel mechanism for defective receptor binding of apolipoprotein E2 in type III hyperlipoproteinemia. Nat Struct Biol.

[38] Wan Ahmad WN, Sakri F, Mokhsin A, Rahman T, Mohd Nasir N, Abdul-Razak S, Md Yasin M, Mohd Ismail A, Ismail Z, Nawawi H (2015). Low serum high density lipoprotein cholesterol concentration is an independent predictor for enhanced inflammation and endothelial activation. PLoS One.

[39] Packard K, Majeed F, Mohiuddin S, Mooss A, Hilleman D, Arouni A (2005). Low high-density lipoprotein cholesterol is associated with impaired endothelial function in Asian Indians. Ethn Dis.

[40] Valappil AV, Chaudhary NV, Praveenkumar R, Gopalakrishnan B, Girija AS (2012). Low cholesterol as a risk factor for primary intracerebral hemorrhage: a case-control study. Ann Indian Acad Neurol.

[41] Zhao D, Zhang Z, Wu G, Wang H, Gao F, Duan X, Lu Y, Wang Z, You D, Qu Y, Song J (2016). Apolipoprotein E gene polymorphism and the risk of subarachnoid hemorrhage: a meta-analysis of case-control studies. Acta Neurochir.

[42] Scicchitano PCM, Maiello M, Modesti PA, Muiesan ML, Novo S (2014). Nutraceuticals and dyslipidaemia: beyond the common therapeutics. J Funct Foods.

